# Illinois State Dental Society

**Published:** 1884-10

**Authors:** 


### ILLINOIS STATE DENTAL SOCIETY.

### TWENTIETH ANNUAL MEETING.

REPORTED EXPRESSLY FOR THE INDEPENDENT PRACTITIONER.

(Continued from page 515.)

### FRIDAY, MAY 16TH.

### DENTAL EDUCATION.

No formal paper was presented under this head, but the subject was opened by Dr. Cushing, who said that the tendency to specialties sometimes made men move in narrow grooves. The true professional man should be as broad as his mental capacity would permit. Dental education should be discussed with reference to the future. He urged higher, more advanced standards in educational requirements.

*Dr. Taft*—Said: We work for the future. The position taken today governs the future. What are the points most to be considered in this connection?

The first is preliminary education. We need a more thorough, comprehensive preliminary education for the future dentist. It is admitted that the educated man is more influential in our profession and in the community at large. He wields a greater influence. The profession in the future will be what you make it now; for the profession is responsible for its own character.

In conclusion, he urged that provision be made for Post-Graduates to carry on their future studies.

On the invitation of a committee composed of Drs. Black and Kitchen, his Excellency Gov. Hamilton visited the society, and made a short address, in which he said that the State of Illinois had done and was still doing much to foster professional education, and that he was in full sympathy with that effort.

Dr. J. J. R. Patrick read a paper on "Irregularities in Human Teeth; or, Dental Teratology."

He insisted, at the outset, that practical education in dentistry is better than theory. The authors of papers in some of the dental journals and in society transactions were continually showing their unlimited confidence in their own ability, based upon limited observation of the different branches of natural science.

The relative proportion of crown and root of teeth is rarely maintained; teeth with long or large crowns having short roots, while those that have low or short crowns have long roots. Great care should be observed in shifting such teeth, for teeth with long roots require more time to place them in a new position, while those having short roots and long crowns, owing to the greater leverage afforded by the crown, are liable to be lifted out of their sockets.

The speaker traced the development of the teeth from the follicular up to the completed stage, illustrating at the same time the coincident development of the maxillary bones. He said the deciduous teeth, on account of their soft and porous nature and the thinness of enamel, are readily permeated by fluids, and for this reason are subject to early decay. The pulp chambers of these teeth being proportionately larger than are those of the permanent set increases the liability to exposure of the pulp in these teeth, and the resulting difficulties that stand in the way of their preservation. He urged the importance of preserving the deciduous teeth, giving as one reason therefor, that if extracted several years before the time for their successors to appear the cells of the extracted teeth would fill with osseous matter and cause the succeeding teeth to diverge from their proper course, and make their exit through either the external or the internal wall of the alveoli.

If teeth are diverted by the mechanical forces of nature, it is evident that they may be brought into their normal positions by mechanical means, intelligently applied. If the diversion was a gradual process, replacement should also be expected to require time for its accomplishment. Until the calcification of the roots of teeth is complete, which in the incisors and canines is not until the tenth or eleventh year of age, the movement of such teeth with a view to mechanical regulation should be postponed until after that age. The condition of the cusps of the incisor teeth may be taken as a fair indication of the condition of the roots. So long as the notches or cusps remain on these teeth the calcification of the roots is incomplete. A similar observation of the canines will serve as a guide to the condition of their roots also. All devices for the shifting of teeth should be simple in design, that they may be easily understood and applied. They should have two points of action; one on teeth that are not to be moved, and one on the tooth that is to be moved. In his opinion the necessity for extraction of teeth to correct irregularity is of rare occurrence, and hence it is best to defer extraction until the case has been thoroughly studied.

Dr. W. B. Ames, of Chicago, read a paper on "Some Applications of Electrolysis in Dentistry."

The uses of electrolysis in medicine are limited to the removal of morbid growths. In dentistry this process, as applied to electrotyping and electro-plating, may be made useful for a variety of purposes. A shell of copper may be thrown upon a plaster model and used in moulding celluloid. Crowns of platinum may be plated with gold, and 18-carat gold plates may be electro-plated with pure metal, and thus corrosion in the mouth prevented. The bleaching of natural teeth by safe chemical process is necessarily slow, yet by the aid of electricity it may be done quickly and with safety, when proper care is exercised. Peroxide of hydrogen is a bleaching agent of great power, as is shown in its effect in bleaching hair, and by means of electrolysis its equivalent can be readily manufactured in the tooth to be bleached.

Ozone is intensified oxygen, and one volume of it is estimated to purify three million volumes of putrid air. Thus it is seen that in the evolution of nascent oxygen or ozone within the putrid mass, it may be quickly and thoroughly disinfected. This may be done within the pulp chamber in the presence of the putrid pulp. By this method of disinfection decomposed pulps may be removed without fear of bad results.

Dr. C. R. E. Koch, of Chicago, read an essay entitled, "The Illinois State Dental Society. What has it accomplished?" Giving a history of the Society from the beginning on July 24, 1865, up to the present time.

During the meeting the society visited the Lincoln homestead. The home is filled with a collection of pictures, publications and other articles which have a historic interest, and which serve to perpetuate the memory of President Lincoln.

Dr. J. G. Templeton, of Pittsburgh, Pa., exhibited an articulating model that contained some novel features, claiming that by its use it is possible for any dentist of ordinary ability to acquire the knowledge and skill in mechanical manipulation that will secure to him such precision and accuracy in articulating entire sets of artificial teeth, that failure in the final result will be of rare occurrence.

The following is a list of the officers of the society for the year ending in May, 1885:

President—H. H. Townsend, Pontiac.
Vice-President—Mark H. Patten, Springfield.
Secretary—J. W. Wassall, 103 State St., Chicago.
Assistant Secretary—P. J. Bester, Chicago.
Treasurer—C. R. Dwight, Danville.
Librarian—J. W. Conway, Mount Carroll.

